# Supplementary material for: Transcriptomic Changes Following Induced De-Masculinisation of Australian Red Claw Crayfish Cherax quadricarinatus
Source: Int J Mol Sci. 2023 Feb 7;24(4):3292. doi: 10.3390/ijms24043292 (PMC9966960; doi:10.3390/ijms24043292)
Supplement: Supplementary file 1 [file ijms-24-03292-s001.zip › Supplementary File S1-dsRNA sequences used in this study.pdf]

Supplementary file S1 - nucleotide sequence of the CDS of Cq-IAG & GFP.

>gi|111182687|gb|DQ851163.1| Cherax quadricarinatus insulin-like androgenic gland factor mRNA, complete cds
ACGCGGGAGGTGCGGCACCAGCAGTGGCCCTCAGCACCCGTCCAGCACAACACCACCACTTCATTTCTTT
GCCTCTTCCCCACTGTGACTGTCCTCCTTCCCACTGTGACTGTCCTCCTTCCCACTGCGACTGTCCTCTT
CCCCATCTGTGACTGTCCTCCTCCCCCATCTGTGACTGTCCTCCTCGCCTATCTGTGACTGTCCTCCTTC
CCACTGTGACTGTCCTCCTCCTCTATCTGTGACTGTCCTCCTACCCATCTGTGACTGTCCTCCTTCCCAC
TGTGACTGTCCTCCTCCTCATCTGTGACTGTCCTCCTCCCCTATCTGTGACTGTCCTCCTCCTCATCTGT
GACTGGCCTCCTCCCCTATCTGTGACTGTCCTCCTCTTCCACTCTTACCTAAAGTCACTATTCTGCTGCT
GGCTGGTTCCCCACCTCATTCAAACTGTCTTATCAGATTATTCAACTTCTAGTTTGTCTGGACGCCAAGT
AATTATCCATTACTCTCGTGGTCTCCGTCACAGCTTTACCTTCTATCAAAGTATAACACTCAACGATGCT
GTTCCAAACATTACTCAACCTGATTTTGGTTGTGGTGGTGAAGCTGCCTCCTCCCTCCGCCTCTTACAGA
GTGGAAAACCTTCTGATTGACTTCGACTGTGGCCACCTGGCGGACACAATGGACAGTATTTGCCGCACCT
ACCAGGAATTTAACGACACCCGAGCGGTGAGGTCGGCCAGAGATGCATCATTTTCTGCCAGTGTCTCCAT
GTATGACCCCGGGAGTAAGATTGCTGTTCGTCAAGTATACCATCCAAGAGGCAGGAAGTTGGGTGTCAAG
TTTACTGTCCCTGATGCCAGGTTGGGTAAGCAGGAGGCGATGACAGTGAGTCGCGAGGCCGCCCATACGT
TTATAAAGACCCAGAACTACAACCGTCGCCGCCGTAACTCAGATACGACAGACAATACAAGCAGCACTAA
CGTTTATGATGAGTGTTGCAGCGAGAAAACATTGAAGACCTGCGTCTTCGATGAGATTGCCCAGTACTGT
GAACAGTTGGAGGACGGAATCTACGTCAGTTCTTGAGGTGGGTGAGCTGGAGGTAGTGACGGTGGCCTCT
TTAACTCCCCTAACCATTATCAAGACACTGACCCGTCACTGCCGATGTTACATTATCAAGACACTGACCC
GTTACTGCCGATGTTACATTGTACTCAGCAGAAACGAGCCTATTGACCACATTTATATCCTATAATTCTT
AATCAAATGTTCGTTTCTTATTATCTTATGTTGGTGTATTAATTTCTGCCTTGTTTGAATCTGTGACGAC
TTGTTTGGTTAAGCACTTTCTGTATTAGTCCAAATTTGCTCTTTTTTTATCATGACTTTCCAGCGCCTTC
CATTAAATAAATTCGTATTGATCAAAAAAAAAAAAAAAAAAAAA

Yellow - start codon.
Green - mature hormone coding sequence (B and A chains)
Underline - 500 nt ORF sequence used for dsRNA synthesis.

>DQ389577.1:10-819 Synthetic construct SS(TOB)-EGFP fusion protein gene, complete cds
ATGGGAAAAATGGCTTCTCTATTTGCCACATTTTTAGTGGTTTTAGTGTCACTTAGCTTAGCACAAACAA
CCCGGGTACCGGTCGCCACCATGGTGAGCAAGGGCGAGGAGCTGTTCACCGGGGTGGTGCCCATCCTGGT
CGAGCTGGACGGCGACGTAAACGGCCACAAGTTCAGCGTGTCCGGCGAGGGCGAGGGCGATGCCACCTAC
GGCAAGCTGACCCTGAAGTTCATCTGCACCACCGGCAAGCTGCCCGTGCCCTGGCCCACCCTCGTGACCA
CCCTGACCTACGGCGTGCAGTGCTTCAGCCGCTACCCCGACCACATGAAGCAGCACGACTTCTTCAAGTC
CGCCATGCCCGAAGGCTACGTCCAGGAGCGCACCATCTTCTTCAAGGACGACGGCAACTACAAGACCCGC
GCCGAGGTGAAGTTCGAGGGCGACACCCTGGTGAACCGCATCGAGCTGAAGGGCATCGACTTCAAGGAGG
ACGGCAACATCCTGGGGCACAAGCTGGAGTACAACTACAACAGCCACAACGTCTATATCATGGCCGACAA
GCAGAAGAACGGCATCAAGGTGAACTTCAAGATCCGCCACAACATCGAGGACGGCAGCGTGCAGCTCGCC
GACCACTACCAGCAGAACACCCCCATCGGCGACGGCCCCGTGCTGCTGCCCGACAACCACTACCTGAGCA
CCCAGTCCGCCCTGAGCAAAGACCCCAACGAGAAGCGCGATCACATGGTCCTGCTGGAGTTCGTGACCGC
CGCCGGGATCACTCTCGGCATGGACGAGCTGTACAAGTAA

Yellow - start codon.
Green – Coding ORF
Underline - 500 nt ORF sequence used for dsRNA synthesis.
